# Using bioprinting and spheroid culture to create a skin model with sweat glands and hair follicles

**DOI:** 10.1093/burnst/tkab013

**Published:** 2021-05-04

**Authors:** Yijie Zhang, Enhejirigala, Bin Yao, Zhao Li, Wei Song, Jianjun Li, Dongzhen Zhu, Yuzhen Wang, Xianlan Duan, Xingyu Yuan, Sha Huang, Xiaobing Fu

**Affiliations:** 1 Research Center for Tissue Repair and Regeneration, Medical Innovation Research Department and the Fourth Medical Center, Chinese PLA General Hospital and PLA Medical College, Beijing 100048, China; 2 PLA Key Laboratory of Tissue Repair and Regenerative Medicine and Beijing Key Research Laboratory of Skin Injury, Repair and Regeneration, Chinese PLA General Hospital and PLA Medical College, Beijing 100853, China; 3 Research Unit of Trauma Care, Tissue Repair and Regeneration, Chinese Academy of Medical Sciences, 2019RU051, Beijing 100048, China; 4 College of Graduate, Tianjin Medical University, Tianjin 300070, China; 5 Institute of Basic Medical Research, Inner Mongolia Medical University, Hohhot 010110, Inner Mongolia, China; 6 The Shenzhen Key Laboratory of Health Sciences and Technology, Graduate School at Shenzhen, Tsinghua University, Shenzhen 518055, Guangdong, China; 7 Department of General Surgery, the Sixth Medical Center, Chinese PLA General Hospital, Beijing 100048, China; 8 Department of Burn and Plastic Surgery, Air Force Hospital of Chinese PLA Central Theater Command, Datong 037000, Shanxi, China; 9 School of Medicine, Nankai University, Tianjin 300071, China

**Keywords:** Skin regeneration, Sweat glands, Hair follicle, 3D bioprinting, Spheroid culture, Skin constructs

## Abstract

**Background:**

Sweat glands (SGs) and hair follicles (HFs) are two important cutaneous appendages that play crucial roles in homeostatic maintenance and thermoregulation, and their interaction is involved in wound healing. SGs can be regenerated from mesenchymal stem cell-laden 3D bioprinted scaffolds, based on our previous studies, whereas regeneration of HFs could not be achieved in the same model. Due to the lack of an *in vitro* model, the underlying molecular mechanism of the interaction between SGs and HFs in regeneration could not be fully understood. The purpose of the present study was to establish an *in vitro* model of skin constructs with SGs and HFs and explore the interaction between these two appendages in regeneration.

**Methods:**

To investigate the interaction effects between SGs and HFs during their regeneration processes, a combined model was created by seeding HF spheroids on 3D printed SG scaffolds. The interaction between SG scaffolds and HF spheroids was detected using RNA expression and immunofluorescence staining. The effects of microenvironmental cues on SG and HF regeneration were analysed by altering seed cell types and plantar dermis homogenate in the scaffold.

**Results:**

According to this model, we overcame the difficulties in simultaneously inducing SG and HF regeneration and explored the interaction effects between SG scaffolds and HF spheroids. Surprisingly, HF spheroids promoted both SG and HF differentiation in SG scaffolds, while SG scaffolds promoted SG differentiation but had little effect on HF potency in HF spheroids. Specifically, microenvironmental factors (plantar dermis homogenate) in SG scaffolds effectively promoted SG and HF genesis in HF spheroids, no matter what the seed cell type in SG scaffolds was, and the promotion effects were persistent.

**Conclusions:**

Our approach elucidated a new model for SG and HF formation *in vitro* and provided an applicable platform to investigate the interaction between SGs and HFs *in vitro*. This platform might facilitate 3D skin constructs with multiple appendages and unveil the spatiotemporal molecular program of multiple appendage regeneration.

Highlights

- Three-dimensional bioprinted skin constructs with sweat glands and hair follicles were successfully established using cell-laden three-dimensional bioprinting and spheroid cultures.
- Through these three-dimensional skin constructs, the interaction effects between sweat glands and hair follicles in regeneration were revealed.
- As a special microenvironmental cue, plantar dermis homogenate promoted the development of sweat glands and hair follicles in regeneration.

## Background

Skin regeneration after trauma and burn is of great significance in the field of plastic surgery. Unlike superficial cutaneous injury, severe wounds can hardly heal spontaneously. However, it is impossible to obtain sufficient skin source for transplantation in patients with large-scale injuries [1–3]. The development of *in vitro* 3D skin tissue constructs facilitates skin reconstructive therapy as well as replaces animal experimentation in pre-clinical testing and advances the capabilities for personalized medicine [4–6]. Unfortunately, with respect to skin production over the past 50 years, we still face many unresolved issues, including physiological structure and function, mechanical strength and readily accessible and reproducible constructs for research and clinical use [7].

In addition to epidermal and dermal components, the inclusion of appendages such as sweat glands (SGs) and hair follicles (HFs) is important for modeling the physiological functions of 3D skin tissue constructs [8–10]. SGs and HFs are important appendages in skin that play crucial roles in the maintenance of body homeostasis, including protection against exogenous stimuli and regulation of body temperature and water–electrolyte metabolism [11,12]. Although *in vitro* fabrication of SGs or HFs is feasible, there is, as of yet, no ideal model to mimic their physiologically symbiotic condition within actual skin [13,14]. This is mainly due to the fact that the molecular program that specifically leads to SG formation is different from those for HFs. *In vivo*, there exist temporal bone morphogenetic protein (BMP)/sonic hedgehog homology (SHH) signal switches in the dermis that allow the emergence of sequential appendages in the developmental process [15]. Nevertheless, it is hard to perfectly reproduce the model of signaling pathways switches *in vitro*. Additionally, the distinct mechanisms underlying the *in vitro* interaction effects between SGs and HFs are crucial for fulfilling the inclusion of multiple appendages in 3D skin tissue constructs that have yet to be modeled.

Cell-laden 3D bioprinting is a cutting-edge technique for regenerative medicine that integrates biophysical, biochemical and biological cues together to promote tissue or organ regeneration [16,17]. 3D bioprinting is also a well-established vehicle for bioactive factors that could provide customized microenvironments promoting lineage differentiation [18,19]. For SG induction, we have previously applied alginate–gelatin bioink and plantar dermis (PD) homogenate to recapitulate the SG microenvironmental niche in 3D cell-printed constructs [13,20]. However, the challenge remains for this technology to produce a microenvironment that allows precise temporal control of signaling pathways *in vitro*. Fortunately, 3D bioprinting can not only function independently, but can also be combined with other techniques for specialized purposes [21]. Thus, in this study, we used spheroid culture technology to induce HF regeneration *in vitro* and then seeded in SG/3D scaffolds. This method allowed us to induce HF and SG formation respectively and concordantly.

Herein, we present an innovative model to effectively explore the interaction effects between SG and HF development. Our approach permits controllable formation of SG and HF in an interdependent and coexisting manner, which mimic the physiological microenvironment within natural skin tissue. Further, this is the first proof-of-concept model to specifically investigate the interaction between SG and HF *in vitro*, which might eventually facilitate the fabrication of 3D skin constructs incorporating multiple appendages.

## Methods

### Study design

**Figure 1.**
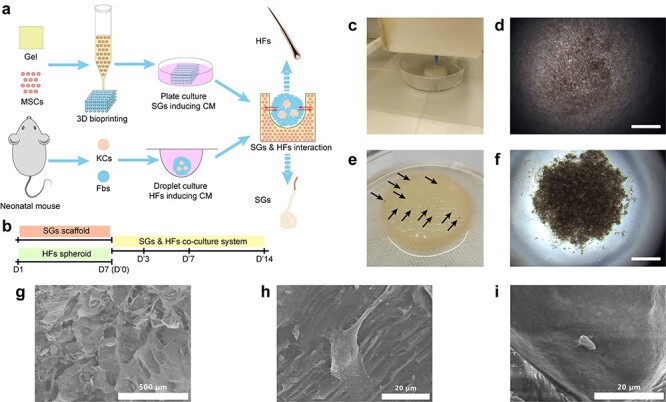
Establishment of 3D skin constructs with multiple appendages. **(a)** Schematic diagram showing the procedure to establish 3D skin constructs *in vitro*. **(b)** Time points used in inducing SGs and HFs separately and SG–HF co-culture. **(c)** 3D bioprinting of SG scaffold. Brightfield imaging of HF spheroid in droplet culture 10 minutes **(d)** and 60 minutes **(f)** after seeding (scale bar, 300 μm). **(e)** Gross imaging of HF seeding on SG scaffold (black arrow shows gross view of HF spheroids seeded on SG scaffolds). **(g)** SEM shows the morphological structure of bioprinted AG constructs (scale bar, 500 μm). **(h)** SEM shows the morphology of MSCs in AG scaffold before SG induction (scale bar, 20 μm). **(i)** SEM shows the morphology of SG-like cells in SG scaffold after SG induction (scale bar, 20 μm). *3D* three-dimensional, *AG* alginate–gelatin gel, *CM* culture medium, *Fbs* fibroblasts, *HFs* hair follicles, *KCs* keratinocytes, *MSCs* mesenchymal stem cells, *SGs* sweat glands, *SEM* scanning electron microscopy

This study was designed to determine whether the *in vitro* co-culture system of 3D skin constructs with SGs and HFs is applicable, and also to explore reciprocal effects between SGs and HFs. The primary steps included the following: (1) bioprinting of a mesenchymal stem cell (MSC)–laden scaffold and SG induction; (2) separation of keratinocytes (KCs) and fibroblasts (Fbs) and droplet culture to form HF spheroids; (3) seeding of HF spheroids onto the SG scaffold; and (4) analysis of the reciprocal effects of SGs and HFs at gene and protein level (Figure 1a, b).

### Animals

The procedures on animals were approved by the Institutional Animal Care and Use Committee of Chinese PLA General Hospital (Beijing, China). C57BL/6 J mice were purchase from SPF Biotechnology Co., Ltd, China and quality controls were performed by Beijing Vital River Laboratory Animal Technology Co., Ltd, China. Enhanced green fluorescent protein (EGFP)-labelled mice were gifted by Prof. Sha Huang and bred in the animal facility of the Fourth Medical Center, Chinese PLA General Hospital under specific pathogen-free conditions.

### Isolation of KCs, Fbs and MSCs

After one-day-old neonatal C57BL/6 J mice were sacrificed, they were sterilized with 75% alcohol. For KCs and Fbs, the back skin was collected and rinsed by phosphate-buffered saline (PBS) twice. To separate epidermis and dermis, the sterilized skin was floated on 0.25% trypsin (Gibco, USA) with the epidermal side up and dermal side down at 4°C for 5 hours, followed by 37°C for 30 minutes. The epidermis was cut into pieces and vortexed to collect KCs. Fbs were collected after the dermis was digested using 0.2% (w/v) type I collagenase (Worthington, USA) at 37°C for 30 minutes. For MSCs, femurs were dissected from the hindlegs and cut into pieces in an anticoagulation buffer (PBS, 10% (w/v); 10 mM ethylene diamine tetraacetic acid, 2% (v/v); and fetal bovine serum). After the anticoagulation buffer was discarded, MSCs were collected following digestion of the femur pieces using 0.25% type I collagenase, supplemented with 20% (v/v) fetal bovine serum, at 37°C for 60 minutes. MSCs were cultured in MesenCult proliferation culture medium (Stemcell Technologies, Canada) and cells at passages 3 to 5 were used.

### Extraction of PDs

Separation of PDs from the plantar dermis was previously described [13]. Briefly, plantar skin dermis was dissected using 0.2% (w/v) Dispase® II (neutral protease, grade II; Roche, Germany) from neonatal plantar skin. A homogenizer was used to dissociate plantar dermis with 4°C PBS at a ratio of 25% (w/v) for 20 minutes to obtain tissue suspension. Following 10,000 *g* centrifugation at 4°C for 10 minutes, the supernatant of the tissue suspension was collected as PD.

### Preparation of MSC-laden 3D bioprinting scaffold and SG induction

Type B gelatin (Sigma-Aldrich, USA) and sodium alginate (Sigma-Aldrich) were dissolved in 0.5 × PBS at 3% and 1% (w/v), respectively, and the final ratio between alginate and gelatin was 1:3. Modified pasteurization was used to sterilize the alginate–gelatin solution. Briefly, the solution was sterilized at 70°C for 30 minutes three times with an interval of 4°C for 10 minutes. After that, the solution was stored at 4°C and incubated at 37°C for 30 minutes before use. The formation of alginate–gelatin gel (AG) was described previously [13]. Briefly, a mixture of 10 mL sterilized alginate–gelatin solution, 200 μL 5 × 10^6^ single-cell suspension of MSCs and 1 mL PD were prepared, followed by gelation at 4°C for 30 minutes. An extrusion-based 3D bioprinter (Bio-Architect PRO, Regenovo, China) was used to bioprint the mixture within a temperature-controlled chamber in which the temperature was settled within the gelation region of gelatin. Cylindrical scaffold was bioprinted layer by layer using nozzles with a 260 μm inner diameter, flow rate of 8–13 mm/s and pressure of 0.05–0.3 MPa. The diameter of the scaffold was 20 mm, with a gap between each printed line of 1.5 mm and totaling 6 layers in height. After the bioprinting process, the printed AG scaffolds were immersed in pre-cooling 2.5% (w/v) calcium chloride for 5 minutes for crosslinking, then washed with Dulbecco’s modified Eagle medium/Ham’s F12 nutrient medium (DMEM/F12, Hyclone, USA) three times. The crosslinked AG scaffold was cultured in an atmosphere of 5% CO_2_ at 37°C with SG induction culture medium (SGCM; Table S1). The medium was replaced with fresh SGCM every three days. Cell morphology was examined and recorded using an automated inverted research microscope (DMI4000 B, Leica, Germany). SG scaffolds would be fully induced in SGCM after 7-days after which the scaffolds would be seeded with HF spheroids.

### HF spheroid formation and seeding on SG scaffolds

After being collected using the procedure described earlier, the KCs and Fbs were mixed at ratio of 1:5–9. The mixture was the seeded into a 20 μL droplet of HF-induction culture medium (HFCM; Table S2) on the lid of a 100 mm dish, with a total cell count of 5 × 10^5^ in each droplet. When the lid was put upside down, the cells sedimented at the bottom of the droplet and aggregated to form HF a spheroid after 24-hour culture. After three days of induction, the HF spheroids were collected in a total volume of 100 μL and seeded onto SG scaffolds from which the culture medium had been discarded. To ensure success of the seeding process, HF spheroids were cultured for 1 hour on medium-free SG scaffolds to guarantee their sedimentation on the bottom of the scaffolds. SG–HF co-culture medium (SG–HFCM; ratio of SGCM:HFCM = 1:1; Table S3) was gently added to the culture dish to immerse the HF-seeded SG scaffold. The medium was replaced with fresh SG–HFCM every three days.

### DiI staining on HF spheroids

The cell mixture of KCs and Fbs was diluted to 1–5 × 10^6^/mL, and then was added with CellTracker CM-DiI dye to the final concentration of 10 μM. The cell mixture was the incubated at 37°C for 15 minutes, avoiding light, and washed with PBS three times. After staining, the cell mixture was seeded onto 20 μL HFCM with a total of 5 × 10^5^ cells in each droplet and droplet seeding process was followed as previous section.

### Quantitative real-time PCR

Total RNA extracted from samples using RNAiso Plus (Takara) was reverse transcribed into template complementary DNA using a PrimeScript RT reagent Kit with gDNA Eraser (Takara, China). Quantitative real-time PCR was performed using TB Green Premix Ex Taq II (Takara, China), according to the manufacturer’s protocol, on QuantStudio 5 system (Applied Biosystems, USA). Specific primers for each gene are listed in Table S4. Each mRNA expression level was normalized against the *Gapdh* mRNA level. Cycling conditions were 95°C for 10 minutes, followed by 45 cycles of 95°C for 10 seconds and 60°C for 30 seconds. Results were expressed by the comparative cycle threshold (CT) method relative to *Gapdh* and finally exported using 2^-ΔΔCT^ method.

### Immunofluorescence staining

The 3D printed structure was harvested and fixed in 4% paraformaldehyde for at least 8 hours. The fixed structure was then dissolved using a 3D lysis solution (55 mM sodium citrate dihydrate, 20 mM ethylene diamine tetraacetic acid and 150 mM NaCl) for 10 minutes. The cells in the 3D structure were collected by centrifugation at 400 *g* for 5 minutes and dropped onto slides. After the cells were attached to slides, slide samples were permeabilized with 0.3% Triton X-100 solution (Sigma-Aldrich, USA) in PBS for 15 minutes. To block nonspecific antigen, slide samples were treated with 5% bovine serum albumin (Sigma-Aldrich, USA) for one hour. Samples were incubated with primary antibodies overnight at 4°C for anti-KRT18 [C-04] (1:300, Abcam, USA, ab668), anti-KRT19 [EP1580Y] (1:300, Abcam, USA, ab52625), anti-KRT17 (1:300, Abcam, USA, ab53707) and anti-ALP (1:300, Abcam, USA, ab95462), and then with secondary antibodies for two hours at room temperature, including CoraLite488 conjugated Affinipure Goat Anti-Mouse IgG (1:200, Proteintech, USA, SA00013–1), CoraLite488 conjugated Affinipure Goat Anti-Rabbit IgG (1:200, Proteintech, USA, SA00013–2), CoraLite594 conjugated Goat Anti-Mouse IgG (1:200, Proteintech, USA, SA00013–3) and CoraLite594 conjugated Goat Anti-Rabbit IgG (1:200, Proteintech, USA, SA00013–4). Sections were counterstained with 4′,6-diamidino-2-phenylinadole (SouthernBiotech, USA) for 15 minutes. All stained slides were scanned using a confocal microscope (TCS SP8 STED, Leica, Germany).

### Scratch assay

MSCs were seeded in 6-well plates at 5 × 10^5^/well. When the cells were fused to 100%, the medium was discarded and washed with PBS. Cells were treated with 2 μg/mL mitomycin (MilliporeSigma, Germany) at 37°C for 2 hours to prevent cell proliferation. Scratches were performed perpendicularly using a pipette tip. The plate was washed at least 3 times to remove detached cells. For the treatment group, HF spheroids were added on the upper side of the transwell chamber. Both groups were cultured with SG–HFCM for 18 hours in the incubator. Images were taken at 0 hours and 18 hours under an optical microscope (CX40, Olympus, Japan).

### Scanning electron microscopy

The skin constructs were freeze-dried using a vacuum freeze dryer (Alpha 2-4 LD Freeze Dryer, Martin Christ, Germany) for 48 hours. Then, the dried skin constructs were coated with gold (20 nm) using an Edwards sputter coater. Finally, the scanning electron microscope (S4800, Hitachi, Japan) was used to detect surface and inner structure.

### Statistical analysis

Statistical analysis was performed using SPSS version 20 (IBM, Inc., USA) or GraphPad Prism 5 (GraphPad Software, Inc., USA). All data are presented as mean ± SD. Student’s *t* test was used to compare two independent samples. One-way Analysis of Variance (ANOVA), followed by Bonferroni’s multiple comparison test, was applied to multiple group comparisons. Differences were considered statistically significant at *p* < 0.05.

## Results

### Establishment of SG and HF co-regeneration using 3D bioprinting and spheroid culture

To establish an *in vitro* 3D model with both SGs and HFs, the pre-induced SGs and HFs were used in this work. Based on our previous work, SGs were induced in 3D bioprinted AG using MSCs as the seed cells and PD as biomimetic microenvironmental cues, which included SG-inducing extracellular matrix and functional proteins (Figure 1c). To form HF spheroids, we seeded a mixture of KCs and Fbs at a ratio of 1:5–9 in droplets that allowed the cells to settle down and pelletize into HF spheroids (Figure 1d, f). After 7-days culture in SGCM and HFCM, the HF spheroids were gently seeded on the surface of the SG scaffolds, where they deposited in the pore spaces of SG scaffolds, forming the SG-HF symbiotic model (Figure 1e). Further, the morphological structures of 3D bioprinted AG scaffolds (Figure 1g), MSC-laden AG gel scaffolds (Figure 1h) and SG scaffolds (Figure 1i) were detected using scanning electron microscopy. Before SG induction, MSCs in AG scaffolds showed extended and stretched morphology (Figure 1h). After SG induction, SG-like cells in the scaffold showed a round and condensate appearance (Figure 1i), which proved the differentiation of MSCs into SG-like cells at the morphological level. KRT 18 and KRT19 are 2 structural marker proteins expressed in luminal cells of SGs [22]. As with HF, KRT17 is the structural marker protein while alkaline phosphatase is the functional protein expressed in the hair shaft [23]. To confirm that the SGs and HFs were induced before seeding, the phenotypes of SGs and HFs were checked by the expression of KRT18 and KRT19 in SGs and KRT17 and alkaline phosphatase in HFs (Figure S1).

### Improvement of SG and HF formation in SG scaffolds by HF spheroids

**Figure 2.**
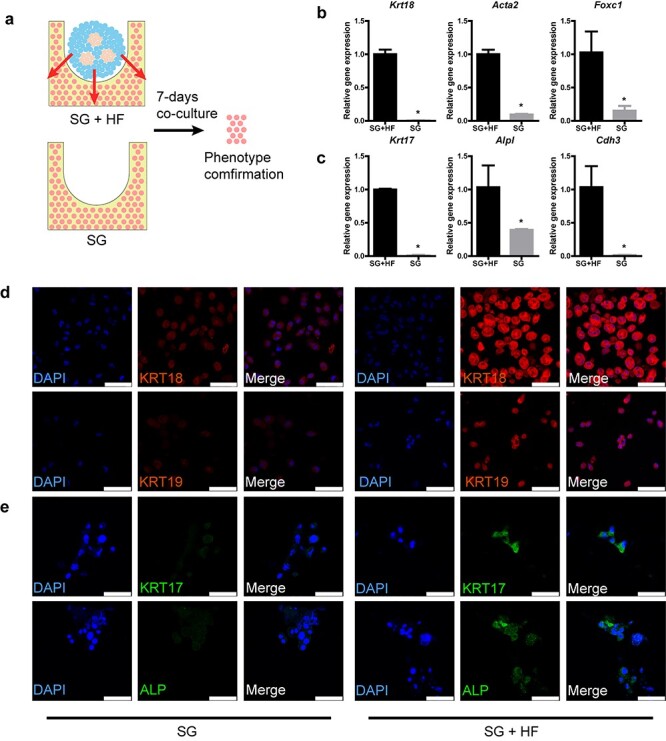
Hair follicle (HF) spheroids promote both sweat gland (SG) and HF genesis in SG scaffolds. **(a)** Schematic diagram showing the procedure to examine the promotion effects of HF spheroids on SG scaffolds. **(b)** Gene expression of SG markers in SG scaffolds after 7-day culture (^*^p < 0.05, n = 3, unpaired Student’s *t* test). **(c)** Gene expression of HF markers in SG scaffolds after 7-day culture (^*^p < 0.05, n = 3, unpaired Student’s *t* test). **(d)** SG-specific markers detected in induced SG scaffolds after 7-day culture (scale bar, 50 μm). **(e)** HF-specific markers detected in induced SG scaffolds after 7-day culture (scale bar, 50 μm). *KRT* cytokeratin. *SG + HF* group means SG scaffolds seeded with HF spheroids, *SG* group means only SG scaffolds

Although the inhibitory interaction effects of SGs and HFs have been illustrated in the developmental model, it is not clear whether this occurs in the *in vitro* regeneration model. The success in establishment of *in vitro* 3D skin constructs with SGs and HFs made it possible for us to examine the crosstalk between appendages in regeneration through this practicable platform. Therefore, the interaction effects between SGs and HFs were verified in this *in vitro* 3D skin construct. To verify the impacts of HF spheroids on SG scaffold, cells in SG scaffolds were extracted for phenotype confirmation (Figure 2a). Structural remodeling plays a pivotal role in organ self-assembly. For SG regeneration, the structural markers of SGs, including the inner layer of the secretory portion (luminal cells, *Krt18+*, *Krt19*+), the outer layer of the support portion (myoepithelium, *Acta2*+) and the sweat ducts (*Foxc1*+) were detected using quantitative real-time PCR and immunofluorescence imaging [11,24,25]. With regard to HF regeneration, *Krt17*, *Cdh3* and *Alpl* are constitutionally expressed in hair progenitor, which contributes to the development and maturation of HFs [23,26,27]. After 7-day co-culture of SG scaffolds and HF spheroids, gene expressions of SGs (*Krt18*, *Acta2*, and *Foxc1*) and HFs (*Krt17*, *Alpl* and *Cdh3*) in the SG scaffold were elevated in the presence of HF spheroids, indicating that the differentiation tendency of SGs and HFs in SG scaffolds was promoted by HF spheroids (Figure 2b, c). In addition, the phenotypes of SG and HF in SG scaffolds were confirmed elevated in fluorescence staining (Figure 2d, e). Notably, different from gene expression, only secretory markers (KRT18 and KRT19) were detected in immunofluorescence imaging of SGs, which revealed that SGs were at an early developmental stage.

### Improvement of SGs in HF spheroids by SG scaffolds

**Figure 3.**
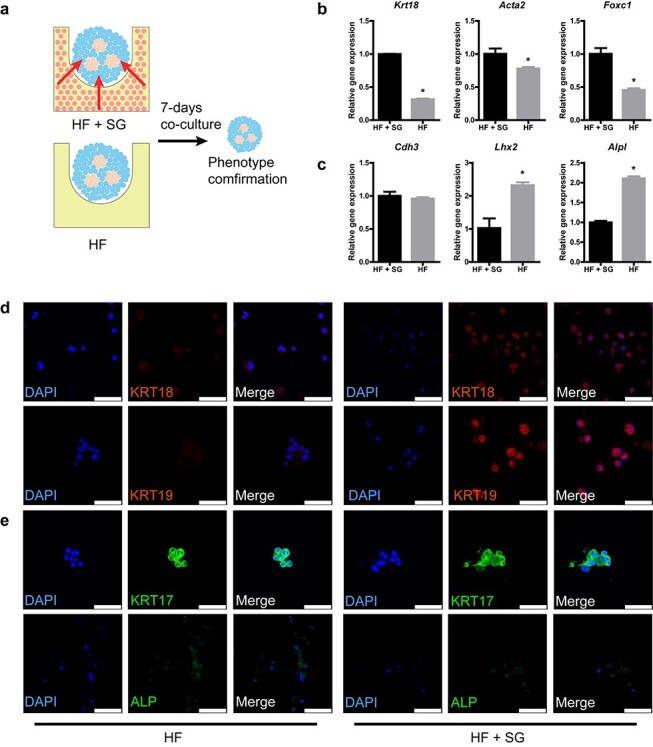
Sweat gland (SG) scaffolds promote SG genesis in hair follicle (HF) spheroids. **(a)** Schematic diagram showing the procedure to exam the promotion effects of SG scaffolds on HF spheroids. **(b)** Gene expression of SG markers in HF spheroids after 7-day culture (^*^p < 0.05, n = 3, unpaired Student’s *t* test). **(c)** Gene expression of HF markers in HF spheroids after 7-day culture (^*^p < 0.05, n = 3, unpaired Student’s *t* test). **(d)** SG-specific markers detected in HF spheroids after 7-day culture (scale bar, 50 μm). **(e)** HF-specific markers detected in HF spheroids after 7-day culture (scale bar, 50 μm). *KRT* cytokerati. *HF+SG* group means HF spheroids seeded on SG scaffolds, *HF* group means only HF spheroids in AG scaffolds

We next examined whether SG scaffolds affected SG and HF formation in HF spheroids. To exclude the effects of scaffolds, we compared HFs cultured in SG scaffolds with those cultured in only AG scaffolds, which is important for providing 3D microenvironmental cues (Figure 3a). After 7-day culture, SG gene expression in HF spheroids seeded in SG scaffolds was increased compared with that in AG scaffolds (Figure 3b), while the gene expression of HF in HF spheroids was partially inhibited (Figure 3c). In the phenotypes of SGs, the elevation of SGs in HF spheroids was confirmed (Figure 3d), while in that of HF, there seemed to be no significant difference (Figure 3e). These results indicate that SG-like cells in SG scaffolds promoted SG differentiation while there was little alteration in HF differentiation in HF spheroids.

### Time sequential tests reveal interaction effects between SG and HF

**Figure 4.**
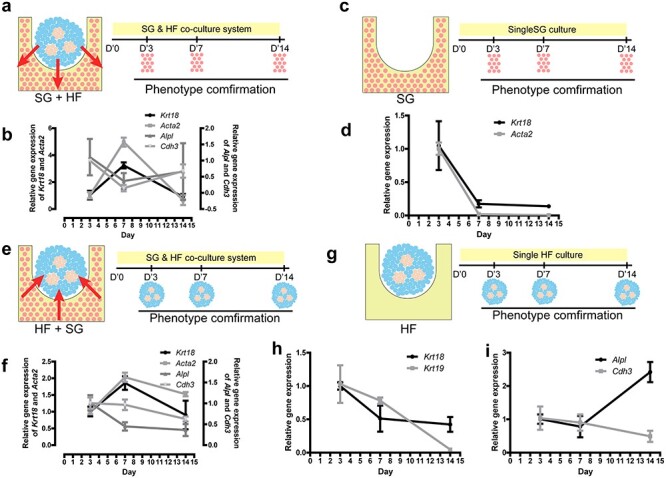
The reciprocal effects between sweat glands (SGs) and hair follicles (HFs) in the time sequential test. **(a)** Schematic diagram showing the impact of HF spheroids on SG scaffolds and the sampling time points. **(b)** Gene expression of SG and HF markers in SG scaffolds after 3-, 7- and 14-day co-culture of HF spheroids seeded on SG scaffolds (n = 3, one-way ANOVA followed by Bonferroni’s multiple comparison test; for statistical significance refer to Table S5). **(c)** Schematic diagram shows single SG scaffolds cultured in a dish and its sampling time points. **(d)** Gene expression of SG markers in SG scaffolds after 3-,7- and 14-day culture without HF spheroids (n = 3, one-way ANOVA followed by Bonferroni’s multiple comparison test; for statistical significance refer to Table S6). **(e)** Schematic diagram shows SG scaffolds impacts on HF spheroids and its sampling time points. **(f)**. Gene expression of SGs and HFs in HF spheroids after 3-, 7- and 14-day culture of HF spheroids seeded on SG scaffolds (n = 3, one-way ANOVA followed by Bonferroni’s multiple comparison test; for statistical significance refer to Table S7). **(g)** Schematic diagram shows single HF spheroids cultured in AG scaffolds and the sampling time points. Gene expression of SGs **(h)** and HFs **(i)** in HF spheroids after 3-, 7- and 14-day culture without SG scaffolds (n = 3, one-way ANOVA followed by Bonferroni’s multiple comparison test; for statistical significance refer to Table S8). *SG+HF* group means SG scaffolds seeded with HF spheroids, *SG group means* only SG scaffolds, *HF+SG* group means HF spheroids seeded on SG scaffolds, *HF* group means only HF spheroids in AG scaffolds

Although the formation of SG and HF seemed elevated after 7-day culture, the true condition in the interaction effects of SGs and HFs in SG scaffold might be covered in only one time point. To test this hypothesis, paralleled to HF spheroids impacts on SG scaffolds, a time sequence test was performed (Figure 4a, c). Interestingly, when taking 3 and 14 days into account, we found an inhibitory interaction effect of SG and HF genesis in SG scaffolds in the presence of HF spheroids (Figure 4b), which is partially due to shifting of BMP/SHH signaling [28]. Nevertheless, in the absence of HF spheroids, the frequency of SG genesis in SG scaffold was decreased in the long term without any increase wave (Figure 4d), and gene expression of HF was undetectable (data not shown). Combined with what is shown in Figure 2, these data hint that HF spheroids might have an induction effect on SG and HF formation in SG scaffold and the promotion of SG and HF in SG scaffolds triggered by HF spheroids might have an inhibitory interaction effect during regeneration.

**Figure 5.**
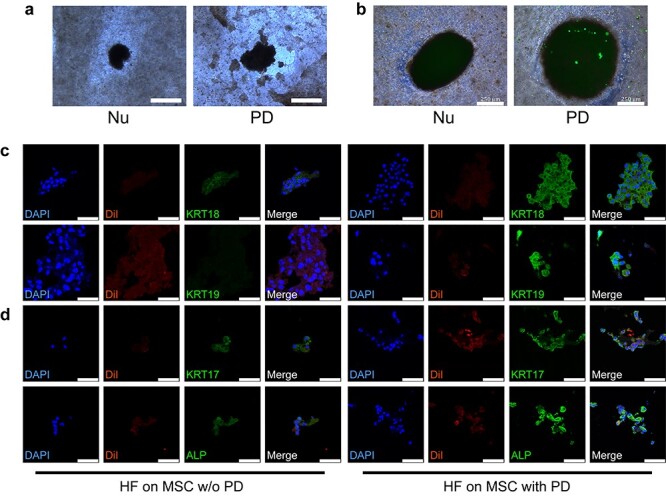
Plantar dermis homogenate (PD) plays vital roles in promotion of sweat gland (SG) and hair follicle (HF) genesis in HF spheroids. **(a)** Brightfield images of HF spheroids and surrounding SG cell mass in SG scaffolds after 14-day culture of HF spheroids seeded on SG scaffolds (scale bar, 500 μm). **(b)** Merged brightfield and green fluorescence images of HF spheroids seeded on SG scaffolds after 14-day culture of HF spheroids seeded on SG scaffolds (green fluorescence expressed by GFP-mesenchymal stem cells (MSCs) in SG scaffolds; scale bar, 250 μm). **(c)** SG-specific markers detected in HF spheroids with MSCs as the seed cells in SG scaffolds after 7-day culture (scale bar, 50 μm). **(d)** HF-specific markers detected in HF spheroids with MSCs as the seed cells in SG scaffolds after 7-day culture (scale bar, 50 μm). *Nu* SG scaffolds w/o PD, *PD* SG scaffolds with PD, *HF on MSC w/o PD* HF spheroids seeded on MSC-laden three-dimensional constructs without PD, *HF on MSC with PD* HF spheroids seeded on MSC-laden three-dimensional constructs with PD. *KRT* cytokerati, *Nu* group means SG scaffolds without PD, PD group means SG scaffolds with PD, *HF on MSC w/o PD group means* HF spheroids seeded on MSC-laden three-dimensional constructs without PD, *HF on MSC with PD* group means HF spheroids seeded on MSC-laden three-dimensional constructs with PD

Similarly, the time sequential test was also performed to explore the long-term effect of SG scaffolds on HF spheroids (Figure 4e, g). Unlike the effects of HF spheroids on SG scaffolds, SGs could only trigger an increased wave of SG genesis in at the 7-day culture point but had no significant effect on HF genesis (Figure 4f). However, in AG scaffolds, HF spheroids has not ascending tendency of SG (Figure 4h), while only the *Alpl* gene expression of HF was increased (Figure 4i), which hinted us that the interaction between SG scaffolds and HF spheroids may be sophisticated.

### PD promotes the interaction and genesis of SGs and HFs in HF spheroids

Microenvironmental cues, including biophysical, biochemical and biological factors, play important roles in tissue and organ regeneration. In our previous work, we identified that PD facilitated SG regeneration in MSC-laden 3D bioprinting [13]. Given that SGs and HFs only coexist in a marginal area of the plantar region, we hypothesized that PD also participated in the crosstalk between SGs and HFs. Therefore, we tested the effect of PD on HF spheroids. Consistent with our previous work, we found SG cells mass in SG scaffold aggregated around HF spheroids in the presence of PD (Figure 5a). To identify the interaction of HF spheroids and SG scaffolds, green fluorescent protein (GFP)-labelled MSCs were used in SG scaffolds. After 14-day co-culture, GFP-labelled SG-like cells were found on HF spheroids in the presence of PD, while there were no GFP-labelled cells on HF spheroids in the absence of PD (Figure 5b). Interestingly, the GFP-labelled cells could hardly be found in the scaffold without PD, a finding which was in accordance with our previous work demonstrating that PD promotes SG regeneration [13]. The weakness of GFP signaling in PD-free scaffold could also be attributed to stretched and extended morphology of undifferentiated MSCs compared to the round and condensate appearance of SG-like cells in SG scaffolds (Figure 1h, i). To exclude the paracrine effect of HF spheroids on MSCs in SG scaffold, we performed a scratch experiment on MSCs in the presence or absence of HF spheroids using a transwell method. After 18-hour co-culture of MSCs and HF spheroids, there was no significant difference in migration (Figure S2), which hinted to us that PD in SG scaffolds might promote the interaction of SG scaffolds and HF spheroids. Given the promoted cell migration from SG scaffolds to HF spheroids, a cell tracker, DiI, was used to exclude this cell migration through specific staining of HF spheroids. In this way, the SG and HF phenotypes in HF spheroids were also enhanced in the presence of PD (Figure 5c, d).

**Figure 6.**
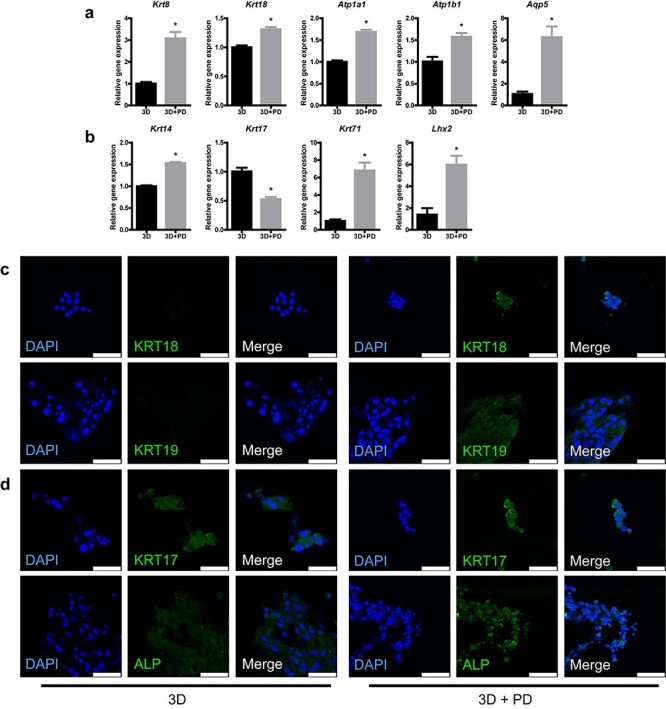
Plantar dermis homogenate (PD) and 3D scaffolds without seed cells promote hair follicle (HF) and sweat gland (SG) genesis. **(a)** Gene expression of SG markers in HF spheroids after 7-day culture (^*^p < 0.05, n = 3, unpaired Student’s *t* test). **(b)** Gene expression of HF markers in HF spheroids after 7-day culture (^*^p < 0.05, n = 3, unpaired Student’s *t* test). **(c)** SG-specific markers detected in HF spheroids on alginate–gelatin gel (AG) or AG-PD scaffolds after 7-day culture (scale bar, 50 μm). **(d)** HF-specific markers detected in HF spheroids on AG or AG-PD scaffolds after 7-day culture (scale bar, 50 μm). *3D* group means HF spheroids seeded on AG scaffolds, *3D+PD group means* HF spheroids seeded on AG-PD scaffolds

We noticed that the different types of seed cells in SG scaffolds would also affect the interaction between SG scaffolds and HF spheroids, MSCs, KCs and Fbs were separately chosen as the seed cell of SG scaffolds. Intriguingly, regardless of the seed cell types in the scaffold, PD exerted a promotional effect on the phenotypes of SG and HF in HF spheroids (Figure 5c, d; Figure S3a, b, c, d). These results gave us a clue that PD in SG scaffolds promoted SG and HF interaction and enhanced the phenotypes of SGs and HFs in HF spheroids independent of the seed cell type in SG scaffolds. The elevation of *Alpl* gene expression in HF spheroids seeded in AG scaffolds could only be partially explained due to the absence of both seed cells and PD in the scaffolds (Figure 4g, i).

### Promotion of SG and HF genesis in HF spheroids by PD and AG scaffolds

Given that the types of seed cells in SG scaffolds were irrelevant to the promotion of PD on SG and HF genesis in HF spheroids, we hypothesized that cell interaction observed previously (Figure 5a, b) has no or less impact on SG and HF formation and that PD–AG scaffolds without seed cells would also have an SG and HF promotional effect on HF spheroids. Surprisingly, unlike the only SG enhancing effect of SG scaffolds on HF spheroids (Figure 3a, b), PD–AG scaffold promoted both SG and HF genesis in HF spheroids. What is more, SG functional genes, namely *Atp1a1*, *Atp1b1* and *Aqp5,* which encode sodium–potassium adenosine triphosphatase (ATPase) and water channel protein, respectively, and HF structural differentiation genes, namely *Krt71,* which encodes HF inner root sheath, were also detected in increased amounts (Figure 6a, b). Similarly, phenotypes of SG and HF were elevated (Figure 6c, d). Overall, the results of cell-free PD–AG scaffolds hinted to us that only PD and AG scaffold promoted SG and HF regeneration in HF spheroids and that PD facilitated this tendency.

## Discussion

Skin appendages are important constituents for fully functional skin [8]. Despite many breakthroughs in skin tissue engineering, an ideal skin model with multiple and fully functional skin appendages is still the ultimate goal of this research area. To date, studies have focused on regenerating skin appendages *in vivo* or a single appendage, such as SG or HF, *in vitro.* Due to the technical limitations of recapitulating the native microenvironment, it is still a challenge to establish a bioengineering skin with multiple appendages [13,14]. 3D bioprinting technology has the potential to better model the cell microenvironment, but precise spatiotemporal control of signaling pathways for both SGs and HFs remains a major bottleneck [29]. To reconcile this, we created a 3D skin construct including both SGs and HFs by combining 3D bioprinting and spheroid culture technology. This approach facilitated fully functional 3D skin tissue constructs with appendages that shared similarities with physiological skin. Experimental use of this *in vitro* model could aid our understanding of the complex relationship between SGs and HFs could facilitate effective recapitulation of the microenvironmental cues required to maintain their symbiotic condition.

Unlike conventional methods for skin engineering, we separately induced SG and HF formation by 3D bioprinting and spheroid culture technology [30]. Moreover, both HF and SG lineage differentiation efficiency were enhanced in SG/3D scaffolds and HF spheroids, which is in agreement with the observed gene and protein expression profiles. These results clearly suggest that the crosstalk between HFs and SGs in the defined 3D skin constructs demonstrates the application potential for investigation of the specific interaction effects of SGs and HFs *in vitro*. Intriguingly, we found that cell-free 3D/PD scaffolds could promote both SG and HF genesis in HF spheroids, which proves the feasibility of simplifying this model to replicate the symbiotic situation and help in understanding complex interactions of HF spheroids in the 3D microenvironment. More importantly, it is in line with the reported literature that inhibition of 3D/PD can result in the reduction of SG growth [14].

Until now, knowledge about the postnatal regeneration of multiple skin appendages has mostly come from studies in animal models [24,31]. Since the animal body offers a physiological environment for skin development, differentiation and morphogenesis of its appendages, SG regeneration remains a major challenge that limits the exploration of their reciprocal communication and potential application in regenerative medicine. This is mainly due to the limited regenerative capacity of SGs and the absence of a SG growth niche in animal skin. Our recent studies addressed this issue by 3D bioprinting to recapitulate microenvironmental cues for SG lineage commitment of MSCs, thereby fully restoring SG structure and function, both *in vitro* and *in vivo* [13,20,32,33]. Additionally, spheroid culture offers a reproducible and efficient tool for HF formation and facilitates structural and functional reconstruction [14]. Despite this success, SG and HF development are both multistage processes consisting of distinct signaling patterns and specific different developmental stages [15]. Our approach—to incorporate engineered HF spheroids into SG/3D scaffolds—represents the first step to circumvent the limitation of engineering the dynamic microenvironment *in vitro*.

Although the interaction effects between SG and HF have been explored in our *in vitro* 3D skin constructs, the complexity of the crosstalk between SGs and HFs is obvious. Microenvironmental cues, such as PD in SG scaffolds, also promote SG genesis in HF spheroids, which hints that the reciprocal effects could be attributed to a modified microenvironment. Lacking morphological and animal results was the most prominent drawback of this work. The elevated SG and HF genesis could be accepted as progenitor because till now it is hard to perfectly manipulate correct microenvironmental cues in multiple appendages regeneration. Given that our strategy significantly enhanced HF and SG lineage differentiation *in vitro*, this model is not representative of a real symbiotic condition of SGs and HFs within native skin, reflecting the limitations of an *in vitro* approach in terms of the culture conditions. This will be addressed in the future by optimized 3D bioprinting constructive design.

## Conclusions

The 3D bioprinted skin construct with SGs and HFs was successfully established by 3D bioprinting and spheroid culture. The mutual inhibitory effect between reconstructed SGs and HFs is revealed in this 3D bioprinted skin constructs indicating the sequential genesis of multiple appendages in regeneration. PD as a microenvironmental cue promoted the development of SGs and HFs in regeneration. In summary, our novel strategy of engineering a 3D skin construct integrating SGs with HFs moves a key step toward a truly functional human skin model *in vitro* and enlightens our understanding of regeneration and interactions between skin appendages and their underlying mechanisms, as well as improving the clinical application of biomimetic 3D skin tissue constructs.

## Supplementary Material

Supplementary_figure_legend_and_table_tkab013

Supplementary_Figure_tkab013

## Abbreviations

3D: three-dimensional; AG: alginate–gelatin gel; Fbs: fibroblasts; HFCM: hair follicle-induction culture medium; HFs: hair follicles; KCs: keratinocytes; KRT: cytokeratin; MSCs: mesenchymal stem cells; PBS: phosphate-buffered saline; PD: plantar dermis homogenate; SGCM: sweat gland–induction culture medium; SG–HFCM: sweat gland–hair follicle co-culture medium; SGs: sweat glands; EGFP: enhanced green fluorescent protein; GFP: green fluorescent protein; DiI: 1,1'-dioctadecyl-3,3,3',3'-tetramethylindocarbocyanine perchlorate; DAPI: 4',6-diamidino-2-phenylindole.
