# Supplementary material for: Using bioprinting and spheroid culture to create a skin model with sweat glands and hair follicles
Source: Burns Trauma. 2021 May 4;9:tkab013. doi: 10.1093/burnst/tkab013 (PMC8240535; doi:10.1093/burnst/tkab013)
Supplement: Supplementary_figure_legend_and_table_tkab013 [file supplementary_figure_legend_and_table_tkab013.docx]

**Supplementary materials**

**Supplementary Figure legend**

**Supplementary Figure 1** SG-specific (KRT18 and KRT19) and HF-specific (KRT17 and ALP) markers detected in induced SG scaffolds and HF spheroids separately before co-cultural seeding. Scale bar: 50 μm. *ALP* alkaline phosphatase , *KRT* cytokeratin

**Supplementary Figure 2** Scratch test of MSC cultured on plate with or without HF spheroids after 18 hrs co-culture. Scale bar: 500 μm. *Ctrl* means control group, *HF* means hair follicle treated group

**Supplementary Figure 3** **a & b**. SG-specific (**a**) and HF-specific (**b**) markers detected in HF spheroids with Fbs as the seed cells in SG scaffolds after 7-days culture. Scale bar: 50 μm. **c & d**. SG-specific (**c**) and HF-specific (**d**) markers detected in HF spheroids with KCs as the seed cells in SG scaffolds after 7-days culture. Scale bar: 50 μm. *KRT* cytokeratin. *HF on Fb w/o PD* group means HF spheroids seeded on Fb-laden three-dimensional constructs without PD, *HF on Fb with PD* group means HF spheroids seeded on Fb-laden three-dimensional constructs with PD, *HF on KC w/o PD* group means HF spheroids seeded on KC-laden three-dimensional constructs without PD, *HF on KC with PD* group means HF spheroids seeded on KC-laden three-dimensional constructs with PD

**Supplementary Tables**

**Table S1 SwG induction culture medium (SwGCM) components.**

| **Reagent** | **Concentration** | **Brand** |
| --- | --- | --- |
| DMEM/F12 | 1 × | Hyclone |
| FBS | 5% (v/v) | Gibco |
| Epidermal growth factor (EGF) | 10 ng/ml | PeproTech |
| 3,3′,5-Triiodo-L-thyronine (T3) | 2 ng/ml | Sigma-Aldrich |
| Hydrocortisone hemisuccinate | 400 ng/ml | Sigma-Aldrich |
| Insulin-transferrin-selenium (ITS) | 1% (v/v) | Gibco |
| Penicillin-streptomycin solution | 1% (v/v) | Solarbio |

**Table S2 HF induction culture medium (HFCM) components.**

| **Reagent** | **Concentration** | **Brand** |
| --- | --- | --- |
| DMEM/F12 | 1 × | Hyclone |
| FBS | 10% (v/v) | Gibco |
| Insulin-transferrin-selenium (ITS) | 1% (v/v) | Gibco |
| 3,3′,5-Triiodo-L-thyronine (T3) | 0.2 nM | Sigma-Aldrich |
| Basic fibroblast growth factor (bFGF) | 20 ng/ml | PeproTech |
| Hydrocortisone hemisuccinate | 2 nM | Sigma-Aldrich |
| Testosterone | 2 nM | Solarbio |
| β-estradiol | 20 nM | Solarbio |
| Progesterone | 20 nM | Solarbio |
| Penicillin-streptomycin solution | 1% (v/v) | Solarbio |

**Table S3 SwG-HF co-coculture medium (SwG-HFCM) components.**

| **Reagent** | **Concentration** | **Brand** |
| --- | --- | --- |
| DMEM/F12 | 1 × | Hyclone |
| FBS | 7.5% (v/v) | Gibco |
| Insulin-transferrin-selenium (ITS) | 1% (v/v) | Gibco |
| 3,3′,5-Triiodo-L-thyronine (T3) | 1.64 nM | Sigma-Aldrich |
| Epidermal growth factor (EGF) | 5 ng/ml | PeproTech |
| Basic fibroblast growth factor (bFGF) | 10 ng/ml | PeproTech |
| Hydrocortisone hemisuccinate | 0.4 μM | Sigma-Aldrich |
| Testosterone | 1 nM | Solarbio |
| β-estradiol | 10 nM | Solarbio |
| Progesterone | 10 nM | Solarbio |
| Penicillin-streptomycin solution | 1% (v/v) | Solarbio |

**Table S4 Primers used in this study**

| **Gene** | **Forward** | **Reverse** |
| --- | --- | --- |
| *Acta2* | GTCCCAGACATCAGGGAGTAA | TCGGATACTTCAGCGTCAGGA |
| *Alpl* | CCAACTCTTTTGTGCCAGAGA | GGCTACATTGGTGTTGAGCTTTT |
| *Aqp5* | AGAAGGAGGTGTGTTCAGTTGC | GCCAGAGTAATGGCCGGAT |
| *Atp1a1* | GGGGTTGGACGAGACAAGTAT | CGGCTCAAATCTGTTCCGTAT |
| *Atp1b1* | GCTGCTAACCATCAGTGAACT | GGGGTCATTAGGACGGAAGGA |
| *Cdh3* | CTGGAGCCGAGCCAAGTTC | GGAGTGCATCGCATCCTTCC |
| *Foxc1* | CAAGACGGAGAACGGTACGTG | GGCTCTCGATTTTGGGCACT |
| *Gaphd* | AGGTCGGTGTGAACGGATTTG | TGTAGACCATGTAGTTGAGGTCA |
| *Lhx2* | CTGTTCCACAGTCTGTCGGG | CAGCAGGTAGTAGCGGTCAG |
| *Krt8* | CAAGGTGGAACTAGAGTCCCG | CTCGTACTGGGCACGAACTTC |
| *Krt14* | AGCGGCAAGAGTGAGATTTCT | CCTCCAGGTTATTCTCCAGGG |
| *Krt17* | ACCATCCGCCAGTTTACCTC | CTACCCAGGCCACTAGCTGA |
| *Krt18* | TCAAGATCATCGAAGACCTGAGG | GCGCATGGCTAGTTCTGTC |
| *Krt71* | GTGGATGCGGCTTATGCCA | CATACTGAGCGCGAACCTCAT |

**Table S5 Statistical significance of Figure 4b**

| **Bonferroni’s test** | ***Krt18*** | | ***Acta2*** | | ***Alpl*** | | ***Cdh3*** | |
| --- | --- | --- | --- | --- | --- | --- | --- | --- |
|  | ***t*** | ***P*** < 0.05 | ***t*** | ***P*** < 0.05 | ***t*** | ***P*** < 0.05 | ***t*** | ***P*** < 0.05 |
| **day1 vs day 7** | 10.52 | Yes | 15.47 | Yes | 1.467 | No | 5.554 | Yes |
| **day1 vs day14** | 0.6278 | No | 1.397 | No | 0.8870 | No | 2.304 | No |
| **day7 vs day14** | 11.15 | Yes | 16.86 | Yes | 0.5795 | No | 3.780 | No |

**Table S6 Statistical significance of Figure 4d**

| **Bonferroni’s test** | ***Krt18*** | | ***Acta2*** | |
| --- | --- | --- | --- | --- |
|  | ***t*** | ***P*** < 0.05 | ***t*** | ***P*** < 0.05 |
| **day1 vs day 7** | 5.003 | Yes | 22.51 | Yes |
| **day1 vs day14** | 5.215 | Yes | 22.89 | Yes |
| **day7 vs day14** | 0.2124 | No | 0.3824 | No |

**Table S7 Statistical significance of Figure 4f**

| **Bonferroni’s test** | ***Krt18*** | | ***Acta2*** | | ***Alpl*** | | ***Cdh3*** | |
| --- | --- | --- | --- | --- | --- | --- | --- | --- |
|  | ***t*** | ***P*** < 0.05 | ***t*** | ***P*** < 0.05 | ***t*** | ***P*** < 0.05 | ***t*** | ***P*** < 0.05 |
| **day1 vs day 7** | 3.650 | Yes | 12.87 | Yes | 4.001 | Yes | 0.4464 | No |
| **day1 vs day14** | 0.4486 | No | 6.517 | Yes | 5.156 | Yes | 4.174 | Yes |
| **day7 vs day14** | 4.098 | Yes | 6.352 | Yes | 0.6107 | No | 3.727 | Yes |

**Table S8 Statistical significance of Figure 4h and 4i**

| **Bonferroni’s test** | ***Krt18*** | | ***Acta2*** | | ***Alpl*** | | ***Cdh3*** | |
| --- | --- | --- | --- | --- | --- | --- | --- | --- |
|  | ***t*** | ***P*** < 0.05 | ***t*** | ***P*** < 0.05 | ***t*** | ***P*** < 0.05 | ***t*** | ***P*** < 0.05 |
| **day1 vs day 7** | 4.481 | Yes | 1.667 | No | 1.052 | No | 0.6164 | No |
| **day1 vs day14** | 5.285 | Yes | 5.926 | Yes | 5.951 | Yes | 2.528 | No |
| **day7 vs day14** | 0.8049 | No | 4.426 | Yes | 6.892 | Yes | 1.911 | No |
