# Supplementary figures and images for: Using bioprinting and spheroid culture to create a skin model with sweat glands and hair follicles
Source: Burns Trauma. 2021 May 4;9:tkab013. doi: 10.1093/burnst/tkab013 (PMC8240535; doi:10.1093/burnst/tkab013)

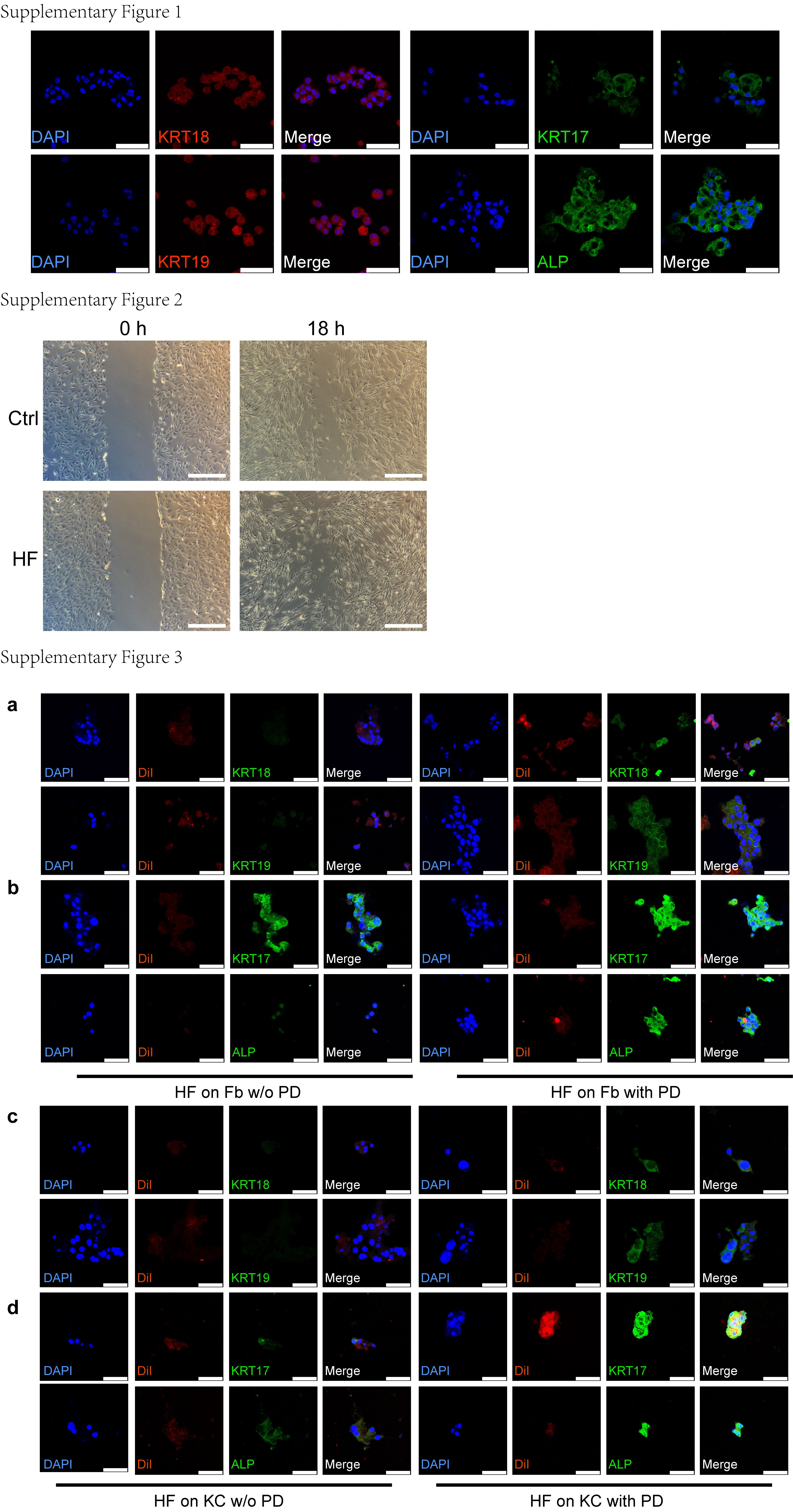

Supplement: Supplementary_Figure_tkab013 [file supplementary_figure_tkab013.jpeg]
